# Hexanoic Acid Treatment Prevents Systemic MNSV Movement in *Cucumis melo* Plants by Priming Callose Deposition Correlating SA and OPDA Accumulation

**DOI:** 10.3389/fpls.2017.01793

**Published:** 2017-10-20

**Authors:** Emma Fernández-Crespo, Jose A. Navarro, Marta Serra-Soriano, Iván Finiti, Pilar García-Agustín, Vicente Pallás, Carmen González-Bosch

**Affiliations:** ^1^Grupo de Bioquímica y Biotecnología, Área de Fisiología Vegetal, Universitat Jaume I, Castellon de la Plana, Spain; ^2^Instituto de Biología Molecular y Celular de Plantas (IBMCP), UPV-CSIC, Valencia, Spain; ^3^Departament de Bioquímica, Instituto de Agroquímica y Tecnología de Alimentos (CSIC), Universitat de València, Valencia, Spain

**Keywords:** MNSV, Cucumis melo, priming by natural compounds, hexanoic acid, OPDA, salicylic acid

## Abstract

Unlike fungal and bacterial diseases, no direct method is available to control viral diseases. The use of resistance-inducing compounds can be an alternative strategy for plant viruses. Here we studied the basal response of melon to *Melon necrotic spot virus* (MNSV) and demonstrated the efficacy of hexanoic acid (Hx) priming, which prevents the virus from systemically spreading. We analysed callose deposition and the hormonal profile and gene expression at the whole plant level. This allowed us to determine hormonal homeostasis in the melon roots, cotyledons, hypocotyls, stems and leaves involved in basal and hexanoic acid-induced resistance (Hx-IR) to MNSV. Our data indicate important roles of salicylic acid (SA), 12-oxo-phytodienoic acid (OPDA), jasmonic-isoleucine, and ferulic acid in both responses to MNSV. The hormonal and metabolites balance, depending on the time and location associated with basal and Hx-IR, demonstrated the reprogramming of plant metabolism in MNSV-inoculated plants. The treatment with both SA and OPDA prior to virus infection significantly reduced MNSV systemic movement by inducing callose deposition. This demonstrates their relevance in Hx-IR against MNSV and a high correlation with callose deposition. Our data also provide valuable evidence to unravel priming mechanisms by natural compounds.

## Introduction

A wide range of responses are induced to avoid infection when a plant recognizes an invading pathogen. The mechanisms by which plants activate host defenses in response to viruses are not completely understood. Plant viruses can interfere with and/or compete for a substantial amount of host resources, which can disrupt host physiology and cause disease (Culver and Padmanabhan, 2007; Pallas and García, 2011; Mandadi and Scholthof, 2013). A large body of evidence has recently shown that virus infection disturbs the delicate hormonal balance that governs plant life by considering mainly the highly relevant involvement of crosstalk among different hormone pathways in antiviral defense (Alazem and Lin, 2015; García and Pallas, 2015; Collum and Culver, 2016). Thus the *Tobacco mosaic virus* (TMV) replicase interacts with and alters the subcellular localisation of several members of the auxin/indole acetic acid (Aux/IAA) protein family (Padmanabhan et al., 2006). Viral interfere with hormone-signaling pathways. Recently, Rodriguez et al. (2014) showed that the expression of the CP of TMV strain Cg, which infects Arabidopsis Cg-CP, stunts plant growth and delays floral transition. Remarkably, Cg-CP expression negatively regulates the SA-mediated defense pathway by establishing DELLA proteins during *Arabidopsis thaliana* viral infection. This suggests that Cg-CP alters the stability of DELLA proteins as a negative modulation mechanism of antiviral defense responses. This effect is similar to that previously reported for the P2 outer capsid protein of the phytoreovirus *Rice dwarf virus*, which interacts with ent-kaurene oxidase, a key factor in the biosynthesis of gibberellins, and causes dwarf symptoms (Zhu et al., 2005).

Plant hormones and reactive oxygen species (ROS) play important roles in regulating transcriptomic changes during stress responses. The three phytohormones that influence the regulation of plant–virus interactions are salicylic acid (SA), jasmonic acid (JA), and abscisic acid (ABA). SA is essential for establishing local and systemic resistance because both SA biosynthesis and signaling are activated when viral effectors by *R* gene products are recognized, which are hallmarks of incompatible interactions (Vlot et al., 2009). In compatible interactions, SA has been observed to improve plant basal immunity by delaying the onset of viral infection and disease establishment (Mayers et al., 2005). So it is not surprising that the inhibition of SA synthesis or SA-dependent defenses would be one strategy that viruses use to enhance infection (Collum and Culver, 2016). JA is also involved in compatible interactions, but seems to act in a phase-specific mode by being induced in early infection stages (Pacheco et al., 2012; García-Marcos et al., 2013). Virus infections generally inhibit JA-induced gene expression. This is particularly relevant in geminivirus infection because both pathogen localisation and jasmonate synthesis preferentially occur in phloem cells, which makes the suppression of the jasmonate response during infection feasible (Hanley-Bowdoin et al., 2013). ABA has been observed to play a defensive role in plant-virus interactions by inhibiting basic β-1,3-glucanase, which is responsible for the degradation of β-1,3-glucan (callose), the product of which is, in turn, deposited on plasmodesmata, and strengthens them against virus movement (Mauch-Mani and Mauch, 2005). Remarkably, JA precursor *cis*-(+)-12-oxo-phytodienoic (OPDA) has been observed to be involved in callose deposition during the activation of defense responses against *Botrytis cinerea* (Scalschi et al., 2015). Some evidence indicates that jasmonates OPDA, JA, and methyl jasmonic acid (MeJA) act as active systemic signaling molecules (Tamogami et al., 2012; Zhu et al., 2014).

On a genome-wide scale, the gene expression analysis in *A. thaliana* has provided relevant information on compatible plant-virus interactions, and has indicated that the transcriptome of host cells undergoes significant reprogramming during infection (Rodrigo et al., 2012).

Oxidative burst is a common event of incompatible and compatible plant–pathogen interactions (Bolwell et al., 1998, 2002). ROS accumulation has also been observed during viral compatible interactions (Inaba et al., 2011; Manacorda et al., 2013), but more studies are needed to clarify the role of ROS accumulation and its relationship with resistance in plant–virus interactions. Decay in antioxidant enzymes, with a consequent increase in ROS, may be necessary to establish infection, replication and spread of the virus (Clarke et al., 2002). Another study into resistant and susceptible cultivars of *Prunus armeniaca*, inoculated with *Plum pox virus* (PPV), have demonstrated that ROS can activate defense genes, and that regulation of antioxidant enzymes can be relevant for determining susceptibility or resistance to plant viruses (Hernández et al., 2006).

Many natural compounds prime plant defenses, including oligosaccharides, glycosides, amides, vitamins, carboxylic acids and aromatic compounds. Among them, hexanoic acid (Hx) is a potent priming agent with a wide range of host plants and pathogens (Aranega-Bou et al., 2014). Treating tomato plant roots with Hx protects them against both the necrotrophic fungus *B. cinerea* (Vicedo et al., 2009) and the hemibiotrophic bacterium *Pseudomonas syringae* pv. tomato (Scalschi et al., 2013). Hx can activate broad-spectrum defenses early by inducing callose deposition, and by activating the SA and oxylipin pathways. Hx can also prime pathogen-specific responses according to the pathogen and its lifestyle, and has an anti-oxidant protective effect (Finiti et al., 2014; Camañes et al., 2015).

Research on priming resistance against viruses by natural compounds is scarce. Some non-natural resistance inducers are effective against viruses, such as (2,1,3)-benzothiadiazole (BTH), a functional analog of SA that protects tobacco against TMV (Friedrich et al., 1996), and tomato plants against *Cucumber mosaic virus* (CMV) (Anfoka, 2000).

In the present work, we studied the basal response and Hx-induced resistance (Hx-IR) of melon plants to *Melon necrotic ringspot virus* (MNSV). This plant virus belongs to the genus *Carmovirus* in the family *Tombusviridae*, and causes systemic necrotic spots on the leaves and streaks on stems of melon, cucumber, and watermelon, as well as occasional plant collapse (“sudden death”) (Hibi and Furuki, 1985). The MNSV genome is a single-stranded RNA molecule with a sense polarity of 4.3 kb that encodes for at least five different proteins (Genovés et al., 2006). The open reading frame (ORF) at the 5′ end terminates in an amber codon that is read through into a second in-frame ORF to yield two proteins involved in replication: p29 and p89. Cell-to-cell viral movement is supported by two proteins, p7A and p7B, encoded by two small centrally located ORFs (Navarro et al., 2006; Genovés et al., 2009; Serra-Soriano et al., 2014). The ORF at the 3′ end encodes coat protein (CP) p42, which is also involved in the systemic transport of the virus and is a symptom determinant. In the present-day, the only resistance that was found in melon against MNSV is controlled by single recessive gene *nsv* (Nieto et al., 2006). As with most plant viruses, MNSV systemically spreads in melon plants through phloem tissue (Gosalvez-Bernal et al., 2008; Pallás et al., 2011). MNSV is transmitted naturally in soil by the zoospores of the chytrid fungus *Olpidium bornovanus* (Lange and Insunza, 1977), when it attaches to the spore’s outer covering (Ohki et al., 2010). Studies previously performed in our laboratory have shown that melon roots act as reservoirs for MNSV (Gosalvez et al., 2003).

The present study aimed to characterize the molecular mechanisms that underlie the basal response and Hx-IR in melon plants inoculated with MNSV. Although hormonal homeostasis has been studied in different plant–virus interactions, most have been carried out at the tissue-specific level, and very few at the whole plant level. We herein investigated the hormonal response in cotyledons, roots, hypocotyls, stems and leaves of MNSV-infected melon plants. We also demonstrated the efficacy of priming agent Hx for preventing virus systemic movement. Our results revealed an interesting correlation between hormone homeostasis and callose deposition in this pathosystem. This study contributes to our understanding of host defenses in response to viruses in non-model species. It also provides valuable evidence to help unravel the complex priming mechanisms performed by natural compounds, and to reveal the contribution of OPDA, SA and pathogen-induced callose deposition in the local response to prevent the virus from spreading.

## Materials and Methods

### Virus Source

The MNSV-Al isolate used herein was originally obtained from field melon plants (*Cucumis melo* L.) collected in the Murcia Region (Spain), and was kindly provided by Dr. F. Botella (Miguel Hernandez University, Alicante, Spain) (Gosalvez et al., 2003). MNSV-infected leaf tissue was homogenized with 30 mM of phosphate buffer (pH 7.0) that contained 20 mM of mercaptoethanol, and the crude extract was used as an inoculum. Subsequently, the virus was purified essentially as described by Díez et al. (1998) for carmoviruses, and was used to propagate infection by serial mechanical transmissions to melon plants (*C. melo* L. “galia”).

### Plant Material and Bioassays

Thirteen inoculation-plus-sampling experiments were run. Each experiment consisted of growing 36 melon plants in 12 different pots (3 plants per pot), of which 10 pots (30 plants) were mechanically inoculated by rubbing purified virus particles on the fully expanded cotyledons of 7- to 9-day-old seedlings before leaves emerged. The other two pots (six plants) were either mock-inoculated or kept healthy to be used as controls.

Bioassays were performed in a climatic chamber with a day/night period of 16/8 h. Temperatures were 25 and 18°C, respectively, with irradiance of 210 μmol photons m^-2^ s^-1^. The mock-inoculated and healthy control plants were similarly treated.

### Hx Treatments

Three experiments were performed to test the effectiveness of Hx in melon plants as an inducer of resistance against MNSV. In the first experiment, Hx was applied to saturation by foliar spray at 16 mM and by soil drench with 1 L of Hx at 4 mM for 3 and 1 days before infection, and 24 h after infection, as a curative treatment. For the second experiment, two sequential Hx treatments were selected (25 mM), applied by soil drench at 72 and 24 h before MNSV inoculation, and with another application at 24 h post-inoculation. The third experiment involved the same experimental template, but no post-inoculation treatment.

### H_2_O_2_ Determination, Microscopy Analysis, and Callose Quantification

Cotyledons were collected for 3′3-diaminobenzidine (DAB) staining at 24 hpi and 5, 12, and 18 dpi. Cotyledons and leaves were cut and placed immediately in 1 mg mL^-1^ of DAB at pH < 3 for 24 h in the dark. They were subsequently discolored in 96% ethanol and rehydrated in distilled water. Callose deposition in response to MNSV infection was quantified from the digital photographs of the aniline blue-stained cotyledons at 24 hpi and 5, 12, and 18 dpi, as described by Flors et al. (2007). Observations were made under a Leica IRB epifluorescence microscope equipped with a Leica DC300F camera (Leica Microsystems^1^). The number of yellow pixels that corresponded to the stained callose was counted with the GNU Image Manipulation program (GIMP^2^). For the double staining of H_2_O_2_ and callose, plant material was stained with DAB and subsequently stained with aniline-blue, as described above.

### Gene Expression Analysis by Quantitative Real-Time Polymerase Chain Reaction (qRT-PCR)

Gene expression by qRT-PCR was performed with the RNA samples extracted from cotyledons and roots using the Total Quick RNA Cells and Tissues kit (E.Z.N.A. Mini kit^3^), according to the manufacturer’s instructions. The melon tissue samples for RNA isolation were collected at 24 hpi and 5 dpi. Highly pure RNA was used for the RT reaction. The RT reaction was performed according to the manufacturer’s instructions for the Omniscript Reverse Transcriptase kit (QIAGEN^4^). The primers employed for the qRT-PCR are listed in Supplementary Table S1. The *Actin* gene expression level was used as an internal housekeeping control. At least three independent experiments were performed to confirm the results. In each experiment, three biological replicates were utilized to generate means and to determine statistical significance.

### Chromatographic Analysis

For the hormonal analysis, fresh material was frozen in liquid N, ground and freeze-dried. Fresh tissue (0.5 g) was immediately homogenized in 2.5 mL of ultrapure water, and 100 ng mL^-1^ of a mixture of internal standards [(^2^H_6_-ABA (to quantify ABA), ^2^H_4_-SA (to quantify SA), dihydrojasmonic acid [to quantify OPDA, JA, and JA-Ile) and propylparaben (to quantify ferulic acid), all acquired from Sigma–Aldrich] were added prior to extraction (Flors et al., 2008). After extraction, a 20-μL aliquot was injected directly into a ultra-high-performance liquid chromatography (UPLC) system. Hormone analyses were carried out in a Waters Alliance 2690 UPLC system (Milford, MA, United States) inside a nucleosil ODS reversed-phase column (100 mm × 2 mm i.d.; 5 μm; Scharlab, Barcelona, Spain^5^). The chromatographic system was interfaced to a Quattro LC (quadrupole–hexapole–quadrupole) mass spectrometer (Micromass^6^). Version 4.1 of the MASSLYNX NT software (Micromass), was used to process the quantitative data from the calibration standards and plant samples. The concentrations of hormones and metabolites were determined in each sample by normalizing the chromatographic area for each compound with the fresh weight of the corresponding sample.

### Hormonal Treatments

For hormonal treatments, plants were grown and inoculated as described above. One day before inoculation, plants were treated by foliar spray with 100 μM of OPDA, 1 mM of SA or 100 μM of OPDA+1 mM SA. OPDA was purchased from Larodan Fine Chemicals^7^ and SA was acquired from Sigma–Aldrich. Plants were harvested and assessed for disease symptoms evaluations.

### Statistical Analysis

A statistical analysis was carried out by a one-way analysis of variance in the Statgraphics-Plus 5 software for Windows V.5 (Statistical Graphics Corp., Rockville, MD, United States). Means were expressed with standard errors and compared by a Fisher’s least-significant difference test at the 95% confidence interval. All the experiments were repeated at least three times.

## Results

### MNSV Infection Leads to Pathogen-Induced Callose and ROS Accumulation on Local and Systemic Tissues

**FIGURE 1:**
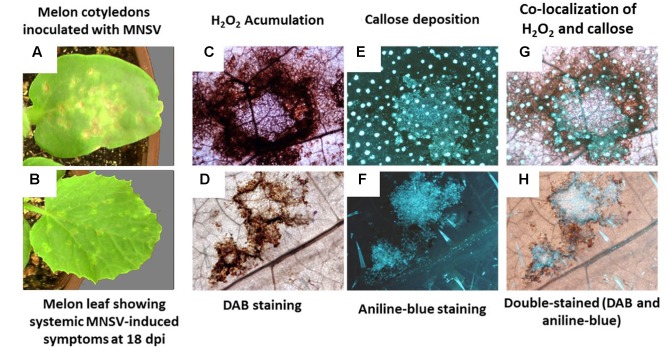
*Melon necrotic spot virus* (MNSV) symptoms in inoculated cotyledons and systemically infected leaves. Localisation of H_2_O_2_ and callose deposition. *Cucumis melo* plants were infected by the mechanical inoculation of fully expanded cotyledons with purified virions of an MNSV-Al isolate. **(A)** Typical MNSV necrotic lesions in the inoculated cotyledons at 5 dpi. **(B)** Necrotic lesions in systemic leaves at 18 dpi. **(C,E)** Localisation of H_2_O_2_ accumulation and callose deposition, respectively, in inoculated cotyledons at 5 dpi. **(G)** Co-localisation of H_2_O_2_ and callose deposition at infection sites. **(D)** Localisation of H_2_O_2_ accumulation and **(F)** callose deposition in the MNSV-infected leaves at 18 dpi. **(H)** Co-localisation of H_2_O_2_ and callose deposition in the systemically infected leaves at 18 dpi.

Upon MNSV inoculation, lesions appeared on cotyledons at 2 or 3 dpi, and had fully developed by 5 dpi (**Figure 1A**). At that time virus systemic movement began and necrotic symptoms were clearly visible on primary leaves at 12 dpi (**Figure 1B**). The histological analyses performed by aniline blue staining demonstrated pathogen-induced callose accumulation at the infection site (**Figure 1E**), but no callose was present in the non-inoculated plants (data not shown). The analysis of hydrogen peroxide (H_2_O_2_) accumulation by 3,3′-diaminobenzidine (DAB) staining showed dark spots around the infection site at 5 dpi, which reflects the oxidative stress associated with this viral infection (**Figure 1C**). The DAB and aniline blue double staining demonstrated that callose and H_2_O_2_ co-located on the lesion area as part of the plant response to MNSV infection (**Figure 1G**). In a late infection stage (18 dpi), ROS and callose accumulated at the infection sites on leaves, which indicates the systemic activation of these defenses in response to MNSV (**Figures 1D,F**). In this case, ROS accumulated to a lesser extent than at the primary infection sites and co-localized with callose (**Figure 1H**).

### Hexanoic Acid-Induced Resistance (Hx-IR) Suppressed MNSV Systemic Movement

**FIGURE 2:**
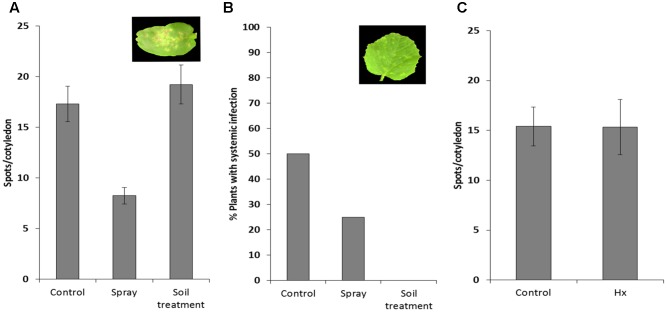
Effect of hexanoic acid on melon plants infected with MNSV. The 1-week-old melon plants were treated with 25 mM of Hx by spray or by soil drench, and were subsequently infected mechanically with the MNSV-Al isolate. Treatments were applied 72 and 24 h before MNSV inoculation, with an additional application 24 h after inoculation. Infection severity was expressed as the number of spots per cotyledon at 5 dpi **(A)** and by counting the number of plants with systemic symptoms at 18 dpi **(B)** Data show a representative experiment repeated three times with similar results. The 1-week-old melon plants were treated with 25 mM Hx, applied by soil drench at 72 and 24 h before MNSV inoculation. Infection severity was expressed as the number of spots per cotyledon at 5 dpi **(C)**. Data show the average of three independent experiments of a pool of 10 plants per experiment ±SE.

We tested the efficacy of natural priming agent Hx as an inducer of resistance against MNSV in melon plants. We set up Hx treatments in melon plants based on previous Hx-IR data in tomato plants against *B. cinerea* and *P. syringae* (Leyva et al., 2008; Vicedo et al., 2009; Scalschi et al., 2013). After checking the different conditions (**Supplementary Figure S1**), we selected the application of two sequential treatments with 25 mM of Hx by soil drench at 72 and 24 h before MNSV inoculation, and another one at 24 h post-inoculation. Disease severity was based on local infection by recounting spots per cotyledon, and on the secondary one by determining the number of plants with systemically symptoms on their leaves. The spray treatment significantly reduced local symptoms and systemic infection (**Figure 2A**). Interestingly, the Hx treatment by soil drench completely blocked MNSV systemic movement (**Figure 2B**), and no significant reduction in local symptoms was noted compared with the untreated plants. We moved on to confirm the prevention of virus systemic spread without the post-inoculation Hx treatment. Hence only the treatments before inoculation were used for further studies (**Figure 2C**). Given the importance of Hx treatment for clearly blocking long-distance viral movement, we searched the biochemical and molecular mechanisms of Hx-IR against MNSV under these experimental conditions. For this purpose, virus entry into the phloem sap was determined by dot blot analysis at 24 hpi and 5 dpi, when disease symptoms still not observed in leaves in control and Hx-treated plants (data not shown). Thus, for further studies we selected control plants in which systemic movement was confirmed at these time points.

### Early Callose Deposition at the Inoculation Site Correlates with the Suppression of MNSV Systemic Spread in Hx-Treated Plants

**FIGURE 3:**
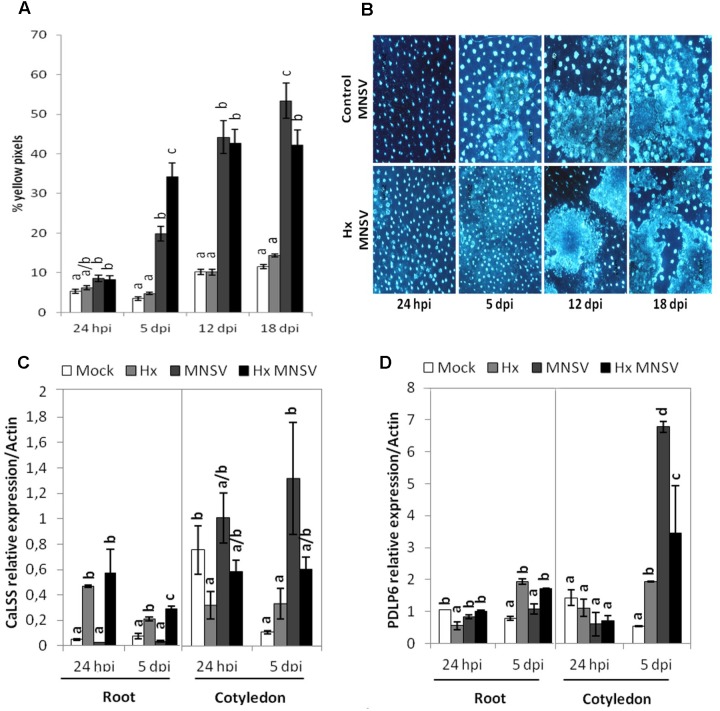
Contribution of callose deposition to Hx-IR against MNSV. Plants were grown and inoculated as described in **Figure 2C** to determine the amount of callose deposition in the control and Hx-treated plants after MNSV infection. Melon cotyledons were sampled at 24 hpi and 5, 12, and 18 dpi, stained with aniline blue and analyzed by epifluorescent microscopy. **(A)** Quantification was performed by determining the percent of yellow pixels in relation to the total pixels in the photographs. Data represent the mean ± SE of three independent experiments with 10 plants per experiment. Letters indicate significant differences between treatments at each time point (*p* < 0.05; least significant difference test). **(B)** Representative pictures of callose deposition in the control and the Hx-treated plants upon MNSV infection (Hx-MNSV). Total RNA was isolated from cotyledons and roots at 24 hpi and 5 dpi, and was converted into cDNA and subjected to a qRT-PCR analysis. The relative level of **(C)** *CaLSS* and **(D)** *PDLP6* was analyzed in the control and treated plants. The results were normalized to the *Actin* gene expression measured in the same samples. Letters indicate significant differences between treatments at each time point (*P* < 0.05; least-significant difference test).

Pathogen-inducible callose deposition significantly increased at 5 dpi in the cotyledons of the Hx-treated plants compared to that in the infected control plants (**Figures 3A,B**). This supports a relevant role of callose priming for Hx-IR in melon plants against MNSV, as previously reported in other pathosystems (Aranega-Bou et al., 2014).

To verify the importance of callose metabolism and the relationship between this polymer accumulation and the block of MNSV movement in the primed plants, the expressions of callose synthase *CalSS* and of *PDLP6*, which encodes a protein involved in callose deposition in plasmodesmata, were analyzed by RT-qPCR. *CalSS* was induced by Hx treatment at 24 hpi in roots. Although both genes were induced by MNSV in cotyledons, no significant changes were observed in their expression by Hx treatment (**Figure 3C**). At 5 dpi in roots, both *CalSS* and *PDLP6* were induced in the Hx-treated and Hx-MNSV plants (**Figures 3C,D**). These findings support the notion that Hx-priming alters the callose metabolism in different tissues, which reflects the relevance of roots in preventing MNSV systemic movement.

### Hx Treatment Reduces ROS Accumulation at the Inoculation Site

**FIGURE 4:**
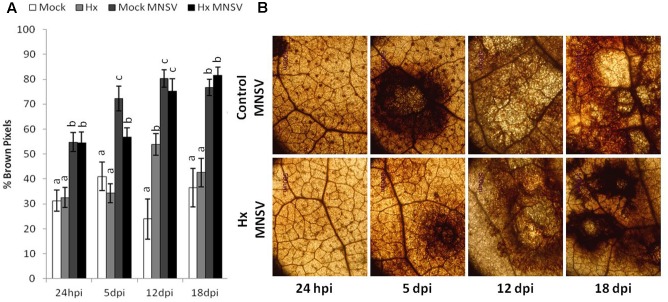
Effect of Hx treatment on oxidative burst after MNSV infection. Plants were grown and inoculated as described in **Figure 3C**. Melon cotyledons were sampled at 24 hpi, 5, 12, and 18 dpi, and H_2_O_2_ accumulation was visualized by DAB staining. **(A)** Quantification was performed by determining the number of brown pixels on digital photographs. Data show average values of the standard errors (*n* = 10) of the relative number of brown or yellow pixels per photograph. Letters indicate significant differences between treatments at each time point (*p* < 0.05; least-significant difference test. **(B)** Representative photographs were taken of H_2_O_2_ accumulation in the mock and Hx-treated plants upon MNSV infection.

The effect of Hx treatment on pathogen-induced ROS production was determined. We observed reduced hydrogen peroxide (H_2_O_2_) accumulation at 5 dpi in the MNSV-inoculated cotyledons compared to the untreated plants (**Figure 4A**). Changes in ROS production were associated with Hx-IR in the tomato plants infected with *B. cinerea*, which alleviated oxidative damage (Aranega-Bou et al., 2014; Finiti et al., 2014). Therefore, it seems that the inducer prevents systemic virus movement by not only priming pathogen-induced callose, buy by also reducing H_2_O_2_ accumulation at the infection site.

### Prevention of MNSV Systemic Movement by Hx-IR Is Associated with Marked Metabolic Reprogramming

**FIGURE 5:**
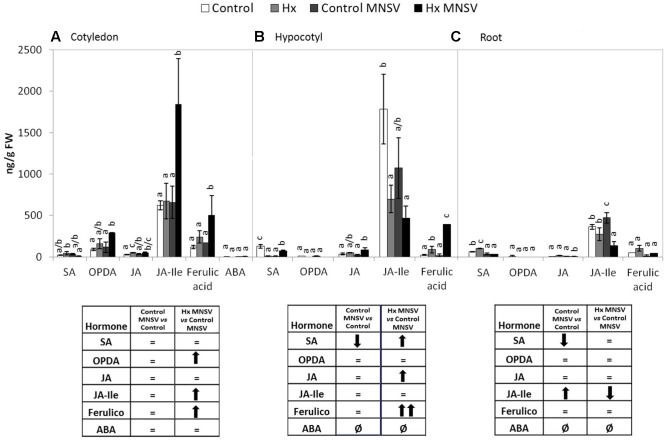
Metabolic profile in the control and Hx-treated plants upon MNSV infection in early infection stages. Plants were grown and inoculated as described in **Figure 2C**. Different tissues were collected at 24 hpi, and the SA, OPDA, JA, JA-Ile, ferulic acid, and ABA levels were determined by UPLC–MS. **(A)** Cotyledon, **(B)** hypocotyl and **(C)** root metabolic profiles. Significant changes are symbolized in a table below the graph: (=) no significant differences; (↑,↓) statistically significant increase or decrease; and (↑↑,↓↓) statistically significant twofold increase or decrease. Data represent the mean ± SE of three independent experiments with 10 plants per experiment. Letters indicate statistically significant differences for each hormone (*p* < 0.05; least significant difference test).

**FIGURE 6:**
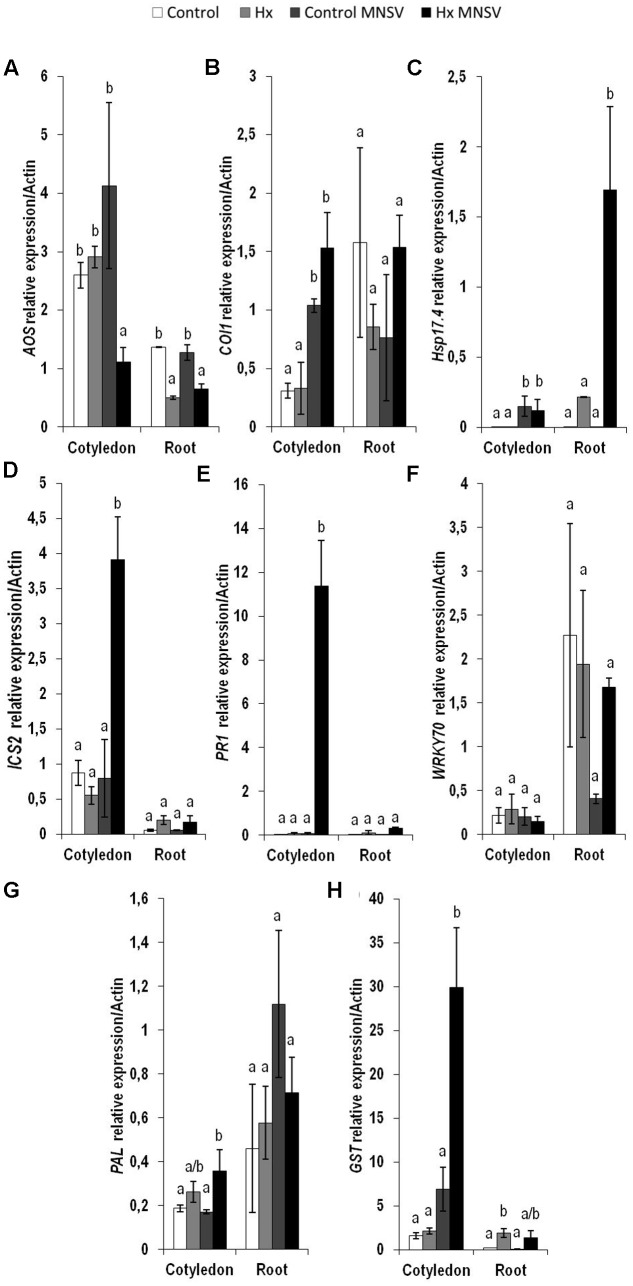
Expression profile of the genes involved in plant defense pathways in the control and Hx-treated plants upon MNSV infection in early infection stages. Plants were grown and inoculated as described in **Figure 2C**. Total RNA was isolated from roots and leaves at 24 hpi, converted into cDNA and subjected to a qRT-PCR analysis. The results were normalized to the *Actin* gene expression level measured in the same sample. The expression levels of genes **(A)** *AOS*, **(B)** *COI1*, **(C)** *Hsp17.4*, **(D)** *ICS2*, **(E)** *PR1* **(F)** *WRKY70*, **(G)** *PAL*, and **(H)** *GST* were analyzed. Data represent the mean ± SE of three independent experiments with 10 plants per experiment. Letters indicate statistically significant differences between treatments at each time point (*p* < 0.05; least significant difference test).

To establish the molecular mechanisms that underlie the prevention of virus spread by Hx-IR, we analyzed the hormonal and metabolite balance of the tissues involved in MNSV movement in melon plants. The most significant biochemical changes noted at 24 hpi occurred in cotyledons and hypocotyls of the Hx-primed and MNSV infected plants. These plants displayed a significant increase in OPDA and JA-Ile in cotyledons, which was associated with ferulic acid accumulation (**Figures 5A,B**). The early basal response (24 hpi) to MNSV was also associated with a slight reduction in SA in roots and hypocotyls, and with an increase in JA-Ile in roots (**Figures 5B,C**). Hx-IR significantly increased ferulic acid in hypocotyls, and slightly increased JA and SA in relation to the basal response. Not many changes were observed in the roots of the Hx-treated plants in this stage, save a slight reduction in JA-Ile (**Figure 5C**). We analyzed the expression of the selected genes in this early infection stage in cotyledons and roots. *ICS2* and *PR1* (SA); *AOS, COI1* and *HSP17.4* (oxylipins, JA, OPDA); *WRKY70* (SA-JA crosstalk); *PAL* (phenylpropanoids); and *GST* (ROS detoxification). The most relevant changes observed in the Hx-treated plants at 24 hpi were the significant induction of *ICS2*, *PR1*, and *GST* in cotyledons, and that of *Hsp 17.4* in roots (**Figure 6**). This reflects the activation of SA- and OPDA-signaling and the antioxidant metabolism as part of Hx-IR. Interestingly, the induction of the SA-pathway occurred with no significant free-SA accumulation in either cotyledons or hypocotyls (**Figure 4B**). The early OPDA accumulation in the infected cotyledons seemed to activate OPDA-dependent signaling pathways in roots, the next tissue involved in virus movement, by higher accumulation of *Hsp17.4* expression (**Figures 5A**, **6C**).

### Metabolic Reprogramming in Both Basal and Hx-IR in Mid- and Late MNSV Infection Stages

**FIGURE 7:**
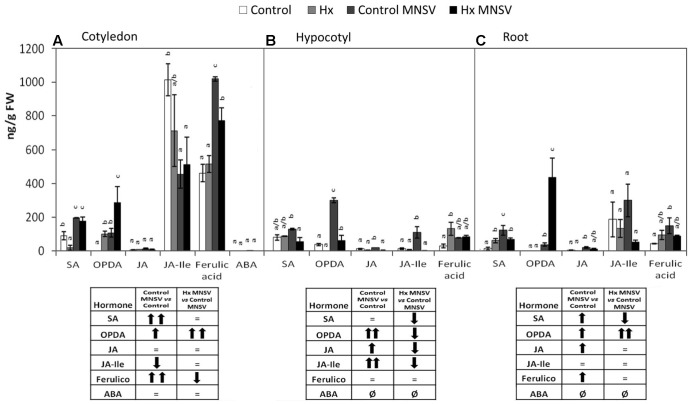
Metabolic profile in the control and Hx-treated plants upon MNSV infection in middle infection stages. Plants were grown and inoculated as described in **Figure 2C**. Different tissues were collected at 5 dpi, and the SA, OPDA, JA, JA-Ile, ferulic acid, and ABA levels were determined by UPLC–MS. **(A)** Cotyledon, **(B)** hypocotyl, and **(C)** root metabolic profiles. Significant changes are symbolized in a table below the graph: (=) no significant differences; (↑,↓) statistically significant increase or decrease; and (↑↑,↓↓) statistically significant twofold increase or decrease. Data represent the mean ± SE of three independent experiments with 10 plants per experiment. Letters indicate statistically significant differences for each hormone (*p* < 0.05; least significant difference test).

**FIGURE 8:**
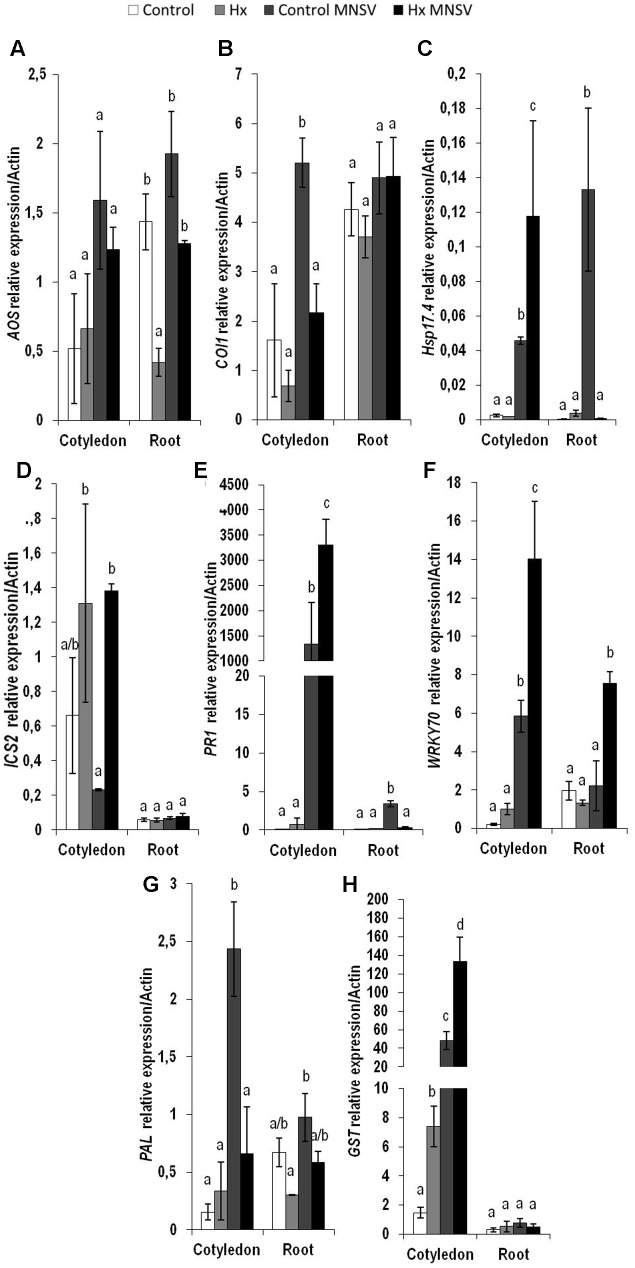
Expression profile of the genes involved in plant defense pathways in the control and Hx-treated plants upon MNSV infection in middle infection stages. Plants were grown and inoculated as described in **Figure 2C**. Total RNA was isolated from roots and leaves at 5 hpi, converted into cDNA and subjected to a qRT-PCR analysis. The results were normalized to the *Actin* gene expression level measured in the same sample. The expression levels of genes **(A)** *AOS*, **(B)** *COI1*, **(C)** *Hsp17.4*, **(D)** *ICS2*, **(E)** *PR1*, **(F)** *WRKY70*, **(G)** *PAL*, and **(H)** *GST* were analyzed. Data represent the mean ± SE of three independent experiments with 10 plants per experiment. Letters indicate statistically significant differences between treatments at each time point (*p* < 0.05; least significant difference test).

In the second infection phase (5 dpi), when MNSV moved to phloem sap, the plant basal response accumulated SA, OPDA, and ferulic acid, and reduced JA-Ile (**Figure 7**). The most significant change associated with Hx-IR in this infection step was the increased OPDA accumulation in cotyledons and roots (**Figures 7A,C**). This reinforces the importance of oxylipin OPDA in plant defense responses. In this stage the gene expression analysis showed that Hx-IR significantly increased *PR1*, *WRKY70*, and *GST* compared to the induction observed in the basal response (**Figure 8**). This once again suggests SA pathway activation and the antioxidant response in the Hx-primed plants upon MNSV infection.

**FIGURE 9:**
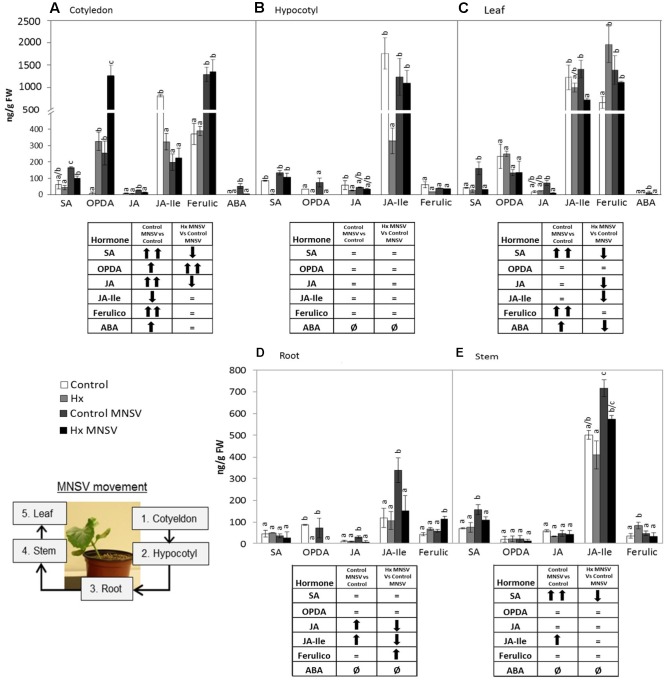
Metabolic profile in the control and Hx-treated plants upon MNSV infection in late infection stages. Plants were grown and inoculated as described in **Figure 2C**. Different tissues were collected at 12 dpi, and the SA, OPDA, JA, JA-Ile, ferulic acid, and ABA levels were determined by UPLC–MS. **(A)** Cotyledon, **(B)** hypocotyl, **(C)** leaf, **(D)** root, and **(E)** stem metabolic contents. Significant changes are symbolized in a table below the graph: (=) no significant differences; (↑,↓) statistically significant increase or decrease; and (↑↑,↓↓) statistically significant twofold increase or decrease. An insert denotes the route of the virus from the inoculation site (cotyledons) to reach systemic tissue (leaves) through the vascular system. Data represent the mean ± SE of three independent experiments with 10 plants per experiment. Letters indicate statistically significant differences for each hormone (*p* < 0.05; least significant difference test).

Finally at 12 dpi when systemic infection was established in the MNSV plants, the most relevant metabolic change in the Hx-primed plants with no systemic virus movement was OPDA accumulation in completely necrotised cotyledons (**Figure 9A**). This contrasts with the metabolic alteration observed in the infected plants that presented significant systemic infection at this point (**Figure 9**), and strongly suggests a key role in the blockage of long-distance viral movement.

### Combined Application of OPDA and SA Reduces MNSV Systemic Spread

The gene expression and hormonal analysis results prompted us to investigate whether an exogenous application of OPDA and/or SA prior to virus infection could reduce MNSV systemic movement in melon plants as Hx treatment did.

**FIGURE 10:**
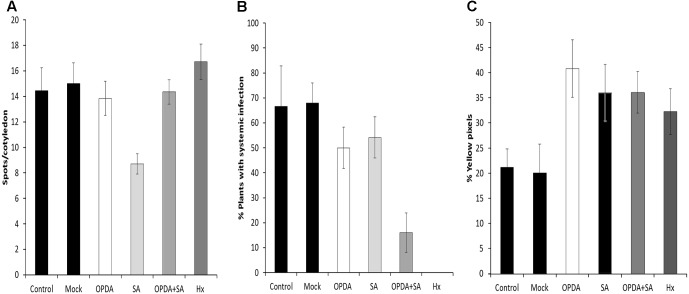
Effect of the OPDA, SA and OPDA+SA treatments on MNSV infection and callose deposition. Infection severity was expressed as the number of spots per cotyledon at 5 dpi **(A)** and by counting the number of plants with systemic symptoms at 18 dpi **(B)** after OPDA, SA, OPDA+SA, and Hx treatments. Melon cotyledons were sampled at 5 dpi, stained with aniline blue and analyzed by epifluorescent microscopy. **(C)** Quantification was performed by determining the percent of yellow pixels in relation to the total pixels in the photographs. Data represent the mean ± SE of three independent experiments with 10 plants per experiment.

For this purpose, OPDA, SA, or OPDA + SA were applied by foliar spray 24 h before infection, and local and systemic disease severity was evaluated (**Figure 10**). At a local level, the SA application lowered the number of spots/cotyledons. However, the OPDA and OPDA+SA treatment did not change local infection compared to the control (**Figure 10A**). Applying OPDA or SA alone resulted in a slighty smaller number of plants showing systemic symptoms (**Figure 10B**). Interestingly, the application of both OPDA and SA significantly reduced viral movement, which lowered the number of melon plants with systemic symptoms to below 16%. The treatment with SA, OPDA and SA+OPDA induced callose deposition in cotyledons 5 days after MNSV inoculation (**Figure 10C**). This demonstrates that accumulation of both OPDA and SA is crucial in the Hx-IR of melon plants to MNSV by contributing to early callose accumulation at the infection site.

## Discussion

The aim of the present study was to gain insights into the basal defense mechanisms of plants against virus attack, and to check the efficacy of priming inducers to control these pathogens. In melon, MNSV causes necrotic brown lesions in inoculated cotyledons. As infection progresses, the virus moves first to roots through the phloem, systemically infects the first leaf, and also produces necrotic spots and necrosis in stems (Gosalvez-Bernal et al., 2008). Upon viral attack, plants defend themselves through several resistance layers that are complementary in defense timing (in the early or late infection steps) and location (in the first infected leaf or in systemic tissues) terms (Nicaise, 2014). From a practical viewpoint, the capability of a plant virus to spread systemically is critical for disease development. Thus the search for chemical compounds to induce systemic resistance or to inhibit viral systemic movement is a feasible alternative to genetic approaches to control viral diseases. We herein analyzed basal and induced melon plant responses against MNSV infection at the whole plant level by including the hormonal profile at three key times of the infection process, specifically at an early time point (24 hpi), in a mid-stage (5 dpi) and in a later stage (12 dpi). We treated melon plants with Hx, a potent natural priming agent effective against bacteria and fungi in different cultures (Aranega-Bou et al., 2014), although its effect on diseases of a viral origin has not yet been tested. After setting up the treatment conditions in melon plants, we found that two soil drench applications of 25 mM Hx before inoculation prevented systemic MNSV movement by confining the virus to the inoculation site. Spray treatment can have a direct effect on the viral cell to cell movement whereas soil drench can affect vascular transport of the virus due to the different hexanoic entry sites. Although it has not been found any receptor for Hx, it seems that roots could play a role in transmitting the protective effect of this natural priming agent in many pathosystems (Aranega-Bou et al., 2014). The promising result obtained prompted us to study Hx-induced resistance (Hx-IR) mechanisms in melon plants in detail. Upon MNSV inoculation, melon plants accumulated callose at the infection site. This was associated with an increase in both the *CalSS* gene, which encodes a callose synthase (Vaten et al., 2011), and the *PDLP6* gene, which encodes a plasmodesmata-localized protein, and which mediates the crosstalk between cell-to-cell communication and innate immunity in Arabidopsis (Lee et al., 2011), at 5 dpi. The Hx-treated plants displayed increased pathogen-induced callose accumulation in cotyledons at 5 dpi compared to that observed in the control plants, which supports the role of callose in Hx-IR (Aranega-Bou et al., 2014). The protective effect against MNSV produced by Hx treatment was similar to the inhibition of plant viral systemic infection by non-toxic concentrations of cadmium (Ghoshoroy et al., 1998). Low levels of Cd ions inhibit virus transport by enhancing callose deposits in the vasculature (Ueki and Citovsky, 2002). Unlike Cd treatment, which seemed to block viral spread into non-inoculated non-vascular tissues, Hx treatment appeared to act in uploading into the phloem. Nevertheless, immune-cytological studies are required to specifically determine the cellular barrier that restricts systemic movement. When we searched the possible transcriptional regulation of this callose priming we found that *CalSS* was induced in cotyledons at 5 dpi but no Hx-induction was observed. However, at 24 hpi and at 5 dpi *CalSS* and *PDLP6* were primed by Hx treatment in roots and slightly increased upon infection at 5 dpi. Despite the importance in plant defense, the regulation of callose biosynthesis at its sites of action within the plant is still limited. In Arabidopsis, it has been found that the timing of stress-induced callose deposition depends on the co-ordination of transport and formation of the callose synthase complex (Ellinger et al., 2013). The penetration resistance to adapted and the non-adapted powdery mildew is based on the transport of the callose synthase *PMR4* to the site of infection, producing increased callose deposition (Naumann et al., 2013). In melon, there is not much information available but our results indicate that Hx-induced *CalSS* and *PDLP6* in roots might have a role on the increased callose deposition at the infection site. Although we cannot discard that other regulators of the callose metabolism be involved in its priming at the infection site, roots are the natural virus entry and play an important role on its movement; hence, further studies will be carried out to clarify this point.

*Melon necrotic spot virus* infection caused oxidative burst at the inoculation site in melon plants. H_2_O_2_ accumulation diminished in the Hx-treated plants compared to the untreated plants at 5 dpi. Hx treatment induced *GST* expression in cotyledons at 24 hpi and 5 dpi compared to the untreated plants. This indicates that Hx treatment could reduce the oxidative damage induced by MNSV infection by activating the antioxidant machinery. This would maintain a lower H_2_O_2_ level, which would suffice to signal plant defenses, but is less toxic for plant tissues. The alleviating effect of oxidative stress by Hx has been demonstrated in tomato against *B. cinerea* and *P. syringae* (Finiti et al., 2014; Camañes et al., 2015). Nováková et al. (2015) have also reported that resistance of *Cucurbita pepo* against *Zucchini yellow mosaic virus* infection is related to a faster accumulation of the proteins involved in ROS detoxification. Our results agree with recent data that support the notion that maintaining a basal level of ROS is essential for normal life (Mittler, 2017).

The metabolic profile of the basal response and Hx-IR to MNSV at the whole plant level, including roots, cotyledons, hypocotyls, stems, and leaves, provided us with clues as to the signaling networks that act in this pathosystem. Our data support a role of both SA and JA signaling pathways in basal and inducible defense against MNSV, and the involvement of OPDA in different plant response steps. At 24 hpi, no significant metabolic changes had occurred in the infected cotyledons, but SA content lowered in roots and hypocotyls. It is important to note that Hx treatment induced the expression of *ICS2*, *PR1*, and *PAL* in cotyledons in an early infection step. Moreover, Hx induced faster and stronger metabolic responses by accumulating OPDA and JA-Ile in cotyledons, and SA and JA in hypocotyls. Hx also primed ferulic accumulation as part of the local response, and also in hypocotyls. This earlier response could help to reinforce the cell wall and to activate local responses that prevent systemic movement because phenolic compounds play a role in cell wall fortification, and display antimicrobial and antioxidant activities (Taheri and Tarighi, 2010, 2011). The higher SA and ferulic acid contents in the Hx-treated hypocotyls, versus those in the control plants, could be used for this very purpose. In compatible interactions, the overexpression of SA biosynthesis genes, or the application of SA or its analogs, often improves the basal response against viruses (Mayers et al., 2005; Peng et al., 2013). SA could significantly restrict the long-distance movement of plant viruses, as several studies have exemplified (see Hipper et al., 2013 for a review).

In higher plants, SA is synthesized by two distinct compartmentalized pathways: the PAL pathway and the ICS pathway (Vlot et al., 2009; Miura and Tada, 2014). In *C. sativus*, the PAL enzymatic pathway contributes to the induced SA production required for chilling tolerance (Dong et al., 2014). Bellés et al. (2008) reported a significant induction of cinnamic acid 4-hydroxylase, an intermediate enzyme of the phenylpropanoid pathway that plays a pivotal role in ferulic acid synthesis in MNSV-infected melon plants. Our findings suggest that *PAL* induction in the cotyledons of MNSV plants at 5 dpi could increase free SA and ferulic acid contents. This late phenylpropanoid pathway activation seems insufficient to induce resistance in melon plants to block MNSV movement and colonization. We speculate that Hx could activate the *ICS2* biosynthetic pathway for SA production, while the *PAL* pathway could increase ferulic accumulation to prevent MNSV movement.

The significantly increased OPDA level by 5 dpi in the cotyledons of the MNSV plants, and its priming at 24 hpi and 5 dpi in the cotyledons of the Hx-MNSV plants, both support its key role in the basal response and Hx-IR (Aranega-Bou et al., 2014). OPDA acts in a JA-independent manner by signaling amino acid biosynthesis and cellular redox homeostasis in stress responses (Park et al., 2013). It is involved in plant responses to pathogens by adopting different infection strategies, such as *B. cinerea* and *P. syringae* (Vicedo et al., 2009; Scalschi et al., 2013). Scalschi et al. (2015) demonstrated a direct role of OPDA in pathogen-callose accumulation, which correlated with resistance to *B. cinerea* in tomato plants. In melon, OPDA-regulated *Hsp17.4* accumulated in cotyledons at 24 hpi, but no changes in its expression were observed in Hx-MNSV. The increase in this gene’s expression in Hx-MNSV plant roots in the first infection step, compared to that in the MNSV plants, supports the role that this signaling pathway plays in this tissue, and highlights the relevance of roots in this pathosystem. Furch et al. (2014) provided data that supported root-to-shoot signaling in *Cucurbita maxima* after flagellin 22 treatment, which rapidly changed the phytohormone concentration in both the phloem and xylem.

The biphasic behavior of JA signaling is noteworthy. JA-Ile content significantly increased at 24 hpi in the Hx-MNSV plant cotyledons. Although *COI1* expression was induced at 24 hpi and 5 dpi in the MNSV plants, it was inhibited at 5 dpi in the Hx-MNSV plants. These findings support the repression of this pathway in later infection stages. At 5 dpi, MNSV cotyledons increased the *WRKY70* transcript levels in Hx-MNSV cotyledons and roots. *WRKY70*, an SA-dependent gene, plays a key role in the SA/JA crosstalk in *A. thaliana* (Li et al., 2006). Interestingly, Ando et al. (2014) reported the crucial role of *WRKY70* in CMV suppression in Arabidopsis through an interaction with the RCY1 disease resistance protein, and by influencing the balance between the SA- and JA-dependent signaling pathways. In the melon-MNSV interaction, *WRKY70* could regulate SA/JA crosstalk, but additional roles cannot be ruled out. Hence Hx-IR seems to prime JA-dependent pathways in the first infection stage; this priming could be reversed by lowering *COI* expression and activating *WRKY70* and *PR1* expressions in later phases. García-Marcos et al. (2013) observed by applying JA treatment to early stages of *Potato virus* Y (PVY) and *Potato virus* X (PVX) double infections enhanced resistance, but applying it later increased susceptibility.

In late infection stages (12 dpi) when MNSV had systemically spread, the SA and ferulic acid levels had significantly risen in leaves. ABA seems to accumulate in this late plant response phase. A significant increase in SA content in stems accompanied a higher JA content. The Hx-treated plants that displayed no systemic infection showed a completely different metabolic profile in leaves, with lower levels of all the analyzed metabolites, and no changes in stems. The cotyledons of the systemically infected plants exhibited significant metabolic changes, such as accumulation of SA, OPDA, JA, ferulic acid, and ABA. This hormonal disruption is common in plants susceptible to viral infection by simultaneously inducing several antagonistic hormones (Alazem and Lin, 2015).

Given the relevant changes in OPDA and SA associated with both basal and Hx-IR against MNSV, and their connection with callose accumulation in other pathosystems (Wang et al., 2013; Scalschi et al., 2015), we treated melon plants with these molecules, either alone or combined, 24 h before MNSV inoculation. The application of 1 mM of SA drastically highly reduced the number of spots/cotyledons, while systemic virus movement slightly reduced in SA or OPDA treatments compared to the untreated plants (**Figure 10B**). However, the melon plants treated with both molecules displayed a significant reduction in the plants that displayed systemic symptoms, which came closer to that observed in the Hx-treated plants (**Figure 9B**). This demonstrates that these hormones have a synergic effect on the resistance of melon plants against MNSV, which could play a prominent role in Hx-IR. Previous data have revealed that exogenous SA applications confer resistance against different viruses, although mechanisms are specific depending on both the host and the infecting virus (Conti et al., 2017). In *Nicotiana benthamiana* plants, SA treatments lowered the coat protein levels of TMV and Potato virus X (PVX) during their compatible interactions (Lee et al., 2011). SA-mediated resistance in *Cucurbita pepo* results from low viral accumulation in inoculated tissues, rather than from systemic movement (Mayers et al., 2005). No previous data on OPDA treatments about virus infection are available, hence our results provide interesting insights into the role of this molecule in plant responses to virus infection. The quantification of pathogen-callose deposition at the infection site in the melon plants treated with SA, OPDA, or SA+OPDA was induced at a similar level to that in the Hx-treated plants in all cases. This supports a role of SA- and OPDA-signaling in the priming of callose against MNSV infection. The fact that the treatment with both molecules was more effective in preventing virus systemic movement indicates that additional mechanisms of callose deposition are essential for blocking systemic MNSV movement by Hx-IR. Ferulic acid accumulation could reinforce the papilla to halt virus movement. In addition, faster antioxidant machinery activation by inducing transcripts like *GST* could also help to maintain optimal ROS levels for proper signaling.

In short, our study provides a global picture of host defenses in response to the virus MNSV in the non-model melon plant. We also demonstrate for the first time the efficacy of natural priming agent Hx to prevent virus systemic movement. The determination of metabolites in different plant tissues in early and late infection stages reveals the relevance of OPDA, SA, and ferulic acid in local responses against MNSV, as well as pathogen-induced callose deposition in both basal and Hx-IR. Treating melon plants with both OPDA and SA significantly reduces virus systemic spread by mediating the increase in pathogen-induced callose deposition. Hence our results contribute to unravel the mechanisms that underlie the priming effect of natural compounds, and demonstrate the potential of these treatments to prevent the virus from spreading.

## Author Contributions

PG-A, VP, and CG-B planned and designed the research. JN, MS-S, and IF performed the experiments. EF-C realized hormonal analysis, qPCR procedures, DAB and callose staining. EF-C analyzing the results and wrote the manuscript with the technical support of CG-B who participated in drafting the manuscript as well. VP and PG-A participated in the correction of manuscript.

## Conflict of Interest Statement

The authors declare that the research was conducted in the absence of any commercial or financial relationships that could be construed as a potential conflict of interest.

## Footnotes

1 http://www.leica-microsystems.com

2 http://www.gimp.org/

3 http://omegabiotek.com

4 http://www.qiagen.com/

5 http://www.scharlab.com

6 http://www.micromass.co.uk

7 http://www.larodan.com

## Supplementary Material

**FIGURE S1 |** Effect of Hx treatment on MNSV disease severity. The 1-week-old melon plants were treated with 16 mM of Hx by foliar spray and by soil drench, and with 4 mM of Hx at 3 days and 1 day before, and at 1 day, after virus inoculation. **(A)** The number of spots per cotyledon at 5 dpi. **(B)** The number of plants with systemic symptoms at 18 dpi.
