# Supplementary figures and images for: Hexanoic Acid Treatment Prevents Systemic MNSV Movement in Cucumis melo Plants by Priming Callose Deposition Correlating SA and OPDA Accumulation
Source: Front Plant Sci. 2017 Oct 20;8:1793. doi: 10.3389/fpls.2017.01793 (PMC5655017; doi:10.3389/fpls.2017.01793)

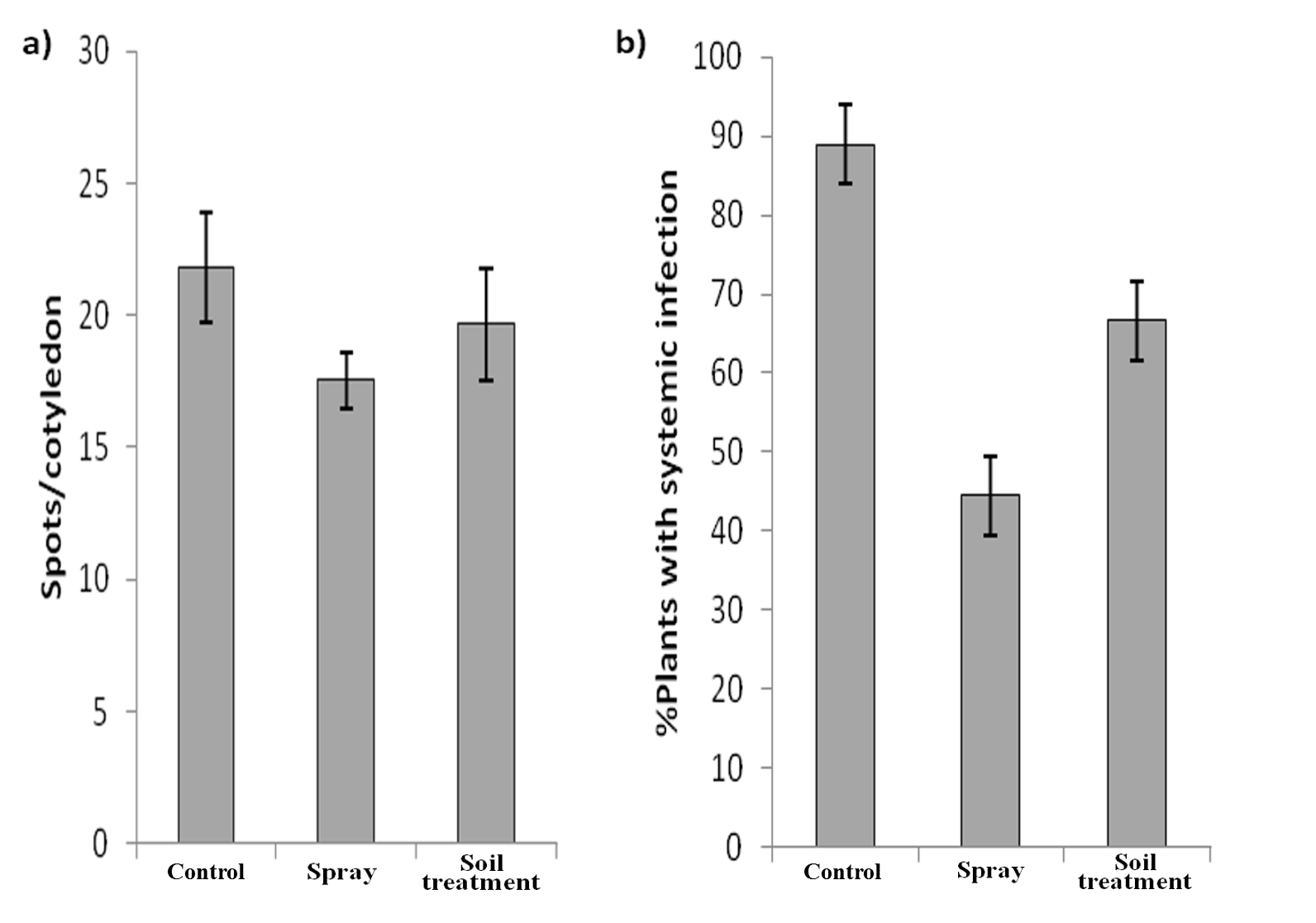

Supplement: Supplementary file 1 [file Image_1.TIF]
