# Supplementary material for: Hexanoic Acid Treatment Prevents Systemic MNSV Movement in Cucumis melo Plants by Priming Callose Deposition Correlating SA and OPDA Accumulation
Source: Front Plant Sci. 2017 Oct 20;8:1793. doi: 10.3389/fpls.2017.01793 (PMC5655017; doi:10.3389/fpls.2017.01793)
Supplement: Supplementary file 2 [file Table_1.DOCX]

**Table S1**. Primers sequences.

| **Gene** | **GenBank accession number** | **Primers** |
| --- | --- | --- |
| *Actin* | XM_008462689.1 | Fwd: 5′- GGAGCTGAGAGATTCCGTTG - 3′  Rev: 5′ - GGTGCAACGACCTTGATTTT -3′ |
| *CalSS* | XM_008462676.1 | Fwd: 5′- tctttgctggcttcaactca - 3′  Rev: 5′- atgcggaaaaagtcaaaacg-3′ |
| *PAL* | XM_008451017.1 | Fwd: 5′- caacttccaagggactccaa - 3′  Rev: 5′ - attgcgatctcagcaccttt -3′ |
| *ISC2* | XM_008454959.1 | Fwd: 5′- tgcttgctgctactgttgct - 3′  Rev: 5′-gcacgttcccagaatgtttt -3′ |
| *AOS* | AF081954.1 | Fwd: 5′- accgtcggttgtacgacttc - 3′  Rev: 5′ - gtcgggtgtggagattcact -3′ |
| COI1 | XM_008464995.1 | Fwd: 5′- agcttgatgcgcttactcgt - 3′  Rev: 5′- gacaatcatacgccggaagt -3′ |
| *JAZ10* | XM_008461681.1 | Fwd: 5′- gcttgatttcttcggtctcg - 3′  Rev: 5′ - ttgggagtaggaggctgaga -3′ |
| *WRKY70* | XM_008458746.1 | Fwd: 5′- caagccacgaagcaagtaca - 3′  Rev: 5′ - ctctcccaccttgattgcat -3′ |
| *GST* | XM_008450384.1 | Fwd: 5′- TTTTGGCCAAGTCCGTTTAG - 3′  Rev: 5′ - GAAATGGGTTTTCCATGGTG-3′ |
| *PDLP6* | XM_008468883.1 | Fwd: 5′- CGCTTCCTTAGACACCTTCG - 3′  Rev: 5′- AGGCCGTAGATGGTGTTTTG -3′ |
